# Rapid Spheroidization Process of S Phase (Al_2_CuMg) in the As-Cast 2024 Al Alloy Induced by High-Energy Electropulsing

**DOI:** 10.3390/ma16216939

**Published:** 2023-10-29

**Authors:** Lai Wei, Xiaofeng Xu, Yang Zhao, Xudong Yan, Yachong Zhou, Yongqiang Yu, Zhicheng Wu

**Affiliations:** 1 Key Laboratory of Automobile Materials, Ministry of Education, Department of Materials Science and Engineering, Jilin University, Changchun 130025, China; 2 Weihai Institute for Bionics, Jilin University, Weihai 264400, China; 3 Key Laboratory of Bionic Engineering, Ministry of Education, College of Biological and Agricultural Engineering, Jilin University, Changchun 130025, China

**Keywords:** spheroidization, 2024 Al alloys, particles, electropulsing treatment

## Abstract

The effect of electropulsing treatment (EPT) on the microstructure of the as-cast 2024 Al alloy at room temperature was investigated. The results show that EPT remarkably accelerated the spheroidizing of second phase (S phase) in the as-cast 2024 Al alloy. The mechanism for rapid spheroidizing of the second phase was proposed based on not only the accelerated dissolution, but also the growth of particles. The morphology and size of the secondary phase could be controlled by changing the cooling speed of the specimen after EPT. Furthermore, the dissolving process of the randomly distributed S phase was recognized as the combination effect of the two basic dissolving ways. Hence, the EPT can be applied to improve the microstructure and properties of the alloys.

## 1. Introduction

Due to its excellent heat resistance, high specific strength, and good formability, 2024 aluminum alloy is widely used in rail transportation, construction, and aerospace [1,2,3,4,5,6]. It plays an important role in energy saving and emission reduction. A large number of second-phase particles are present in 2024 aluminum alloy owing to the high content of alloying elements. The second-phase particle is recognized as an effective factor in slowing the grain growth of polycrystalline materials [7]. As obstacles to the motion of grain boundaries, second-phase particles can effectively retard grain growth [8]. As a result, the morphology, distribution, and size of secondary phases have a significant effect on the microstructure and properties of the alloys. In the past decades, many assumptions upon the Zener theory were established to describe the relationship between grain growth and second-phase particles [9], and parts of previous work focused on the effect of the second-phase morphology on mechanical behavior [10]. Usually, the smaller and spherical second phase is more effective in pinning grain boundaries, which is beneficial not only for the plasticity, but also the strength [8,10,11,12,13,14]. However, although the conventional heat treatment can meet the target, the long-term high temperature may produce a coarse microstructure.

Electropulsing treatment (EPT), an instantaneous high-energy input method, is extensively applied in many fields [15]. EPT is characterized by high efficiency and energy saving, which makes it a promising method in metals processing. Many previous studies show that EPT has an important effect on recrystallization and phase transformations [16,17,18,19]. Hou et al. [20] found that pulsed current could achieve rapid recrystallization of Ti-6Al-4V alloys that are produced by equal channel angular pressing (ECAP), realizing optimizing the strength and plasticity of the material. Shen et al. [21] compared the effects of electric pulse annealing and conventional heat treatment on the mechanical properties of heavy cold-rolling pure Al and found that the pulsed current can achieve the recovery process of pure Al in a shorter duration, and the grains almost did not undergo coarsening. Waryoba et al. [22] used in-situ transmission electron microscopy to study the effect of high-density pulsed current on defects within additively manufactured Ti-6Al-4V alloys. They proved that the electron wind effect generated by high-density pulsed current can reduce/eliminate defects. Yang et al. [23] investigated the effect of high-voltage pulsed current on the microstructure and microhardness of selective laser-melted (SLM) Ti-6Al-4V alloy. They proved that a high-voltage pulsed current can promote the phase transformation of the alloy and refine the lamellar grains, thus increasing the microhardness.

In recent years, it was indicated that the EPT could accelerate the spheroidizing of the precipitation phase and dissolution processes in the Mg alloy. The mechanism for the dissolution of the precipitated particles was recognized as the decreased thermodynamic barrier in the phase transformation process [24,25]. Our recent studies also demonstrated that electropulsing treatment promotes rapid spheroidization of grains in Ti-6Al-4V alloy [26]. However, few investigations were conducted on discussing the process and mechanism of the second-phase spheroidization under EPT. In this paper, we used EPT to treat the as-cast 2024 Al alloy to study the morphology of the second phase (Al_2_CuMg). Scanning electron microscopy (SEM) and X-ray diffraction (XRD) results revealed that the second-phase occurs solution and spheroidzation after EPT. Moreover, the ANSYS software was used to analyze the solution of the second-phase. The finding of this pa-per may be helpful in understanding the shape evolution of second-phase during the EPT process.

## 2. Materials and Methods

**Figure 1:**
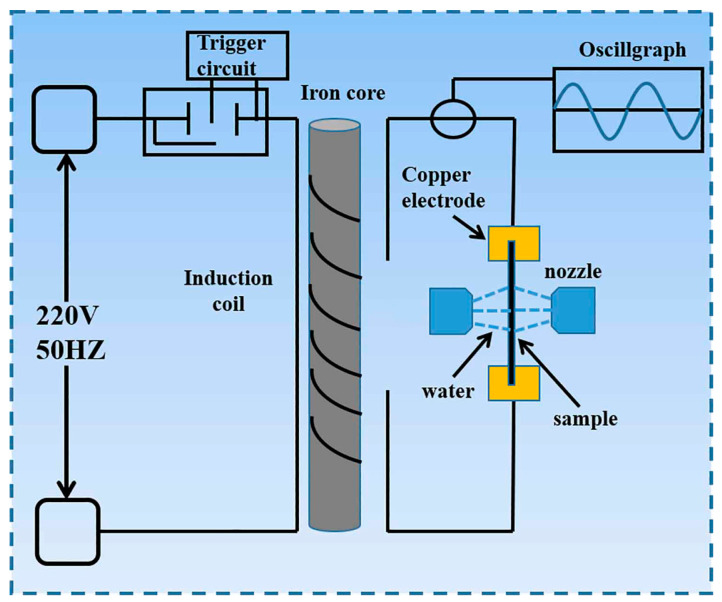
Schematic diagram of the self-made electropulsing generator.

The commercial as-cast aluminum alloy 2024 (4.42 wt.% Cu, 1.49 wt.% Mg, 0.51 wt.% Mn, and balance Al) was used in this study. The chemical composition of the alloy was proven by energy-dispersive X-ray spectroscopy (EDS) data. The sampling position is inside the middle of the ingot. The ingot was cut and polished into pieces (50 mm × 10 mm × 2 mm). The samples were divided into three groups. The as-cast sample (AC) was not subject to any treatment and was employed as the reference sample. The sample treated with electropulsing and water cooled to room temperature was named EPT + WQ. The sample treated with electropulsing and air cooled to room temperature was named EPT + AQ. The oxidized film on the surface of the sample was polished off before EPT, and subsequently, the sample was placed on a copper electrode and fixed with a clamp. The pulse current output was controlled by a computer. The EPT was performed by a self-made electropulsing generator, which could generate an AC pulse current with a 50 Hz frequency [10]. In this study, the current density was optimized to 200 MA/m^2^ with a duration of 240 ms. The self-made electropulsing generator is shown in Figure 1.

The morphology of the phases was investigated with the use of scanning electron microscopy (SEM, Carl Zeiss JSM-5310,Oberkochen, Germany). The SEM specimens were prepared through conventional mechanical polishing and followed by etching with Keller reagent (2 mL HF, 3 mL HCl, 5 mL HNO_3_, and 190 mL water). The phases and dissolution process were identified by X-ray diffraction (XRD) using a Rigaku-D/Max 2500PC/Japan diffractometer with Cu Kα radiation.

The current passage through the precipitated phase was simulated using ANSYS software. In this study, we abstract the second phase as an ellipsoid to represent its morphology. Firstly, the ellipsoidal model was drawn using CATIA software and imported into ANSYS software. The waveform of the current was set to be sinusoidal in ANSYS software, the value of the current was the maximum current measured (200 MA/m^2^), and the duration in the solution setup was the time required for one cycle of the wave (20 ms). It should be noted that to study the dissolution process of grains with different orientations when electropulsing treatment is applied, the second phase with two orientations of length direction (perpendicular to the current direction/parallel to the current direction) was selected. For more on modeling in ANSYS, refer to the citation [27].

## 3. Results and Discussion

**Figure 2:**
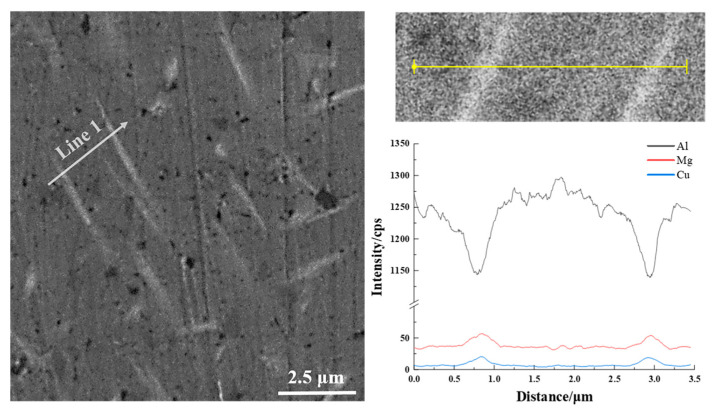
The one-dimensional element profiles of as-cast 2024 Al alloy.

**Figure 3:**
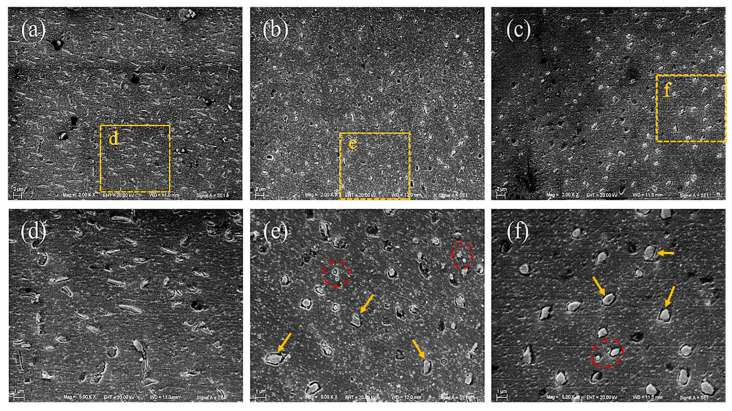
SEM micrographs of specimens under different conditions: (**a**,**d**) AC, (**b**,**e**) EPT + AQ, and (**c**,**f**) EPT + WQ; (**d**–**f**) are the magnification images of (**a**–**c**). The yellow arrows and red dashed areas represent the spherical S phases produced by different dissolution mechanisms, respectively.

**Figure 4:**
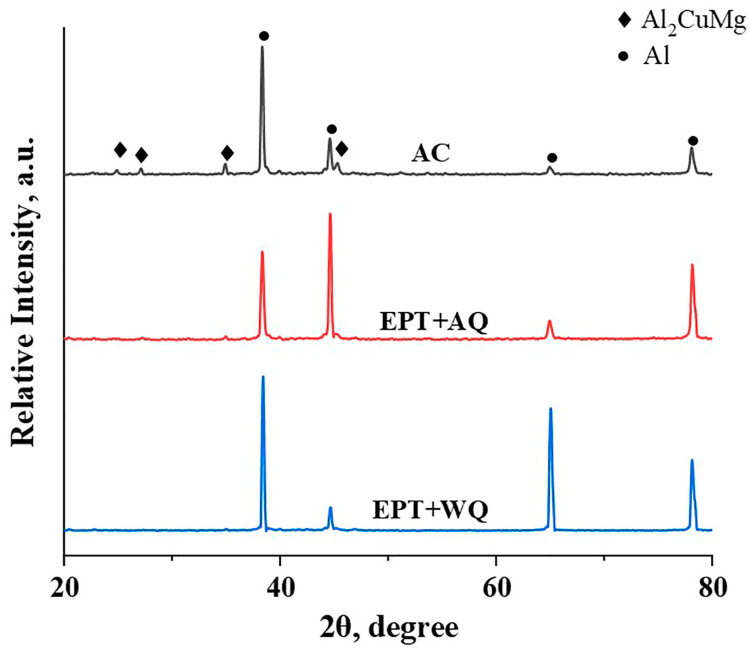
XRD patterns for samples at various conditions.

The primary microstructures of as-cast 2024 Al alloy are the typical as-cast phases in Al-Cu-Mg alloy: the matrix is the α-Al and the particles are identified as S phase (Al_2_CuMg) by EDX (Figure 2). The morphology change of the secondary phases inside the grains was examined by SEM, as shown in Figure 3. The S phase in the as-cast sample has a rod-like shape (Figure 3a,d). The S phases in EPT + WQ (Figure 3b,e) and EPT + AQ (Figure 3c,f) became spherical shapes from strips. The size of the EPT + WQ particles is smaller than that of EPT + AQ, but the EPT + AQ has a more smooth interface and regular shape compared with EPT + WQ. Furthermore, the S phases in the EPT samples can be divided into two categories: comparatively larger-sized S phases that are farther apart (e.g., the area marked by yellow arrows in Figure 3e,f) and smaller-sized S phases that are nearer together (e.g., the area marked by red dashed lines in Figure 3e,f). Comparison of Figure 3a–c can reveal that the proportion of the S phase decreases in the EPT samples (EPT + AC and EPT + WQ), which suggests that the S phase undergoes dissolution after EPT. To prove the dissolution of the S phase, the samples in different states were analyzed by XRD. Figure 4 presents XRD patterns of the samples at different conditions (AC, EPT + AQ, EPT + WQ). The AC sample consists of mainly two phases, namely, α-Al and Al_2_CuMg. As shown in Figure 4, The XRD peaks of the Al_2_CuMg phase in the EPT + AQ and EPT + WQ samples weakened and nearly vanished. This implies that the EPT makes parts of the Al_2_CuMg phase dissolve in the matrix, which is in agreement with the SEM result (Figure 3).

In this study, as a result of the Joule heating effect during electropulsing, the temperature rise can be described as [28]:

(1) $$\Delta T={\rho j}_{e}^{2}(C_{\rho }d{)}^{-1}t_{c}$$

where *ρ* is the resistivity, *Cρ* is the specific heat, d is the density, and *t_c_* is the duration of discharging. Typical conditions were as follows: *ρ* = 4.4 × 10^−8^ Ωm; *Cρ* = 875 J/kg·K; and *d* = 2.78 × 10 kg/m^2^. Based on the parameters, the calculated value of Δ*T* is 174 K. So, *T* = Δ*T* + *T_0_* = 467 K, where *T*_0_ (293 K) is room temperature. It is obvious that the temperature is not high enough to make the second phase dissolve in the matrix, but the effect of the temperature will be discussed in the section below. According to classical thermodynamics, previous studies indicated that the electric current in the conductor results in an additional energy term to the total Gibbs free energy of the system, which plays an important role in the effect of EPT on the solid solution temperature of the second phase. In this study, the main phases are *α*-Al (*α* phase) and S phase (*β* phase), so the phase transformation is expressed as *α* + *β* → *α*′; when there is non-EPT, the energy barrier can be described by

ΔG_0_ = G_α′_ − G_α+β,_ (2)

where *G_α_*_′_ is the molar free energy of *α*’ phase and *G_α_*_+*β*_ the molar free energy of *α* + *β*. While under EPT, the energy change can be described by

ΔG^EPT^ = G_0_ + G_e,_ (3)

where Δ*G_e_* is an energy change due to a change in the distribution of the current in the formation of a nucleus, and when the electric conductivity of the *α* phase is higher than that of O phase, Δ*G_e_* < 0 [15]. It was concluded that *T_e_^EPT^* < T*_e_*, where *T_e_* is the solvus temperature of the S phase in the phase diagram, *T_e_^EPT^* is the dissolving temperature under EPT, and *T_e_* can be determined by Δ*G*_0_ = 0 [15,29], implying that EPT substantially decreased the solid solution temperature of the second phase by decreasing the thermodynamic barrier, compared with conventional heat treatment. When the sample is under EPT, the temperature reaches or exceeds *T_e_^EPT^*, Δ*G^EPT^* < 0, so the secondary phases dissolve in the matrix. Hence, although it does not make the temperature as high as conventional heat treatment, the EPT dramatically accelerated the dissolving of the second phase into a matrix via the coupling of the thermal and athermal effects of EPT [15,25,30]. Furthermore, compared with the traditional equilibrium solid solution treatment [31,32,33,34], the electropulsing treatment significantly reduces the duration required for solid solution treatment, which is significant for energy saving and efficient. While the EPT is completed, the dissolving temperature of the second phase changes back to the *T_e_*, and the temperature of the sample begins declining, Δ*G^EPT^* > 0, so the dissolving process ends.

On account of the relationship between atom diffusion and temperature:

(4) $$D_{1}=D_{0}exp(-\frac{Q}{RT})$$

where *D*_0_ is the diffusion pre-exponential factor, *Q* is the activation energy, and *R* is the gas constant [35]. As is known, the temperature and the lattice diffusion coefficient are improved in EPT. When the temperature of the specimen is raised in a short time, the atoms can obtain large amounts of energy and the diffusion of atoms can act easily [30,36]. Furthermore, with the aid of EPT, the atomic flux is sufficient to accelerate diffusional phase transformation in a very short period.

**Figure 5:**
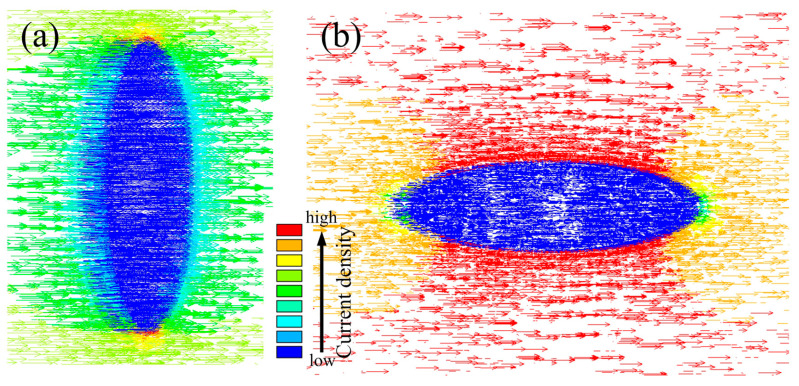
Qualitative numerical simulation result of the current density distribution when the major axis orientation of the S phase is (**a**) perpendicular to the current direction and (**b**) parallel to the current direction.

Figure 5 shows the qualitative numerical simulation result of the current density distribution when the electric current passes through an S phase (which is simplified to ellipsoid) with the ANSYS software. Figure 5a shows the current distribution when the major axis of the S phase is perpendicular to the current direction. It is found that the current density is high at the two end portions; where the temperature will rise rapidly, moreover, the higher current density makes the stronger effect of electromigration [37]. Hence, the two end portions will be the first parts that dissolve in the matrix, a dissolution process that reduces the aspect ratio of the second phase, making it closer to a spherical shape. This dissolution mechanism produces an S phase consistent with the region marked by the yellow arrow in Figure 3. For the same reason, the dissolving starts at the middle part when the current orientation is parallel to the S phase major axis direction, as shown in Figure 5b. Due to the rapid dissolution of the middle part of the S phase, the rod-like S phase split into two separated smaller S phases, which in turn reduces the aspect ratio of the S phase. This mechanism produces the S phase corresponding to the region marked by the red dashed line in Figure 3. For the randomly distributed phases, the mechanism for spheroidizing and the dissolution process is based on the combination effect of the above-mentioned two ways. In addition, the thermal and compression fields inside the material remain after EPT. Under thermal and compression fields, the particles will further undergo the spheroidization and growth process. Currently, there are four mechanisms of grain spheroidization: termination migration, edge spheroidization, cylinderization, and boundary splitting [38,39]. Each mechanism has its corresponding conditions for occurrence. In this study, the as-cast sample dislocation density is low. Therefore, the termination migration mechanism is more likely to occur. Termination migration is a spheroidization process dominated by diffusion [40]. Due to the large curvature of the end of the particles and the small curvature of the smooth region in the center, a potential energy difference is created between the end and the flat interface, and this energy gradient causes the elements at the end of the particles to migrate towards the smooth region to complete the spheroidization process [41]. In addition, the spheroidization process is accompanied by growth, and with the combined effect of both, the S phase will have higher sphericity.

**Figure 6:**
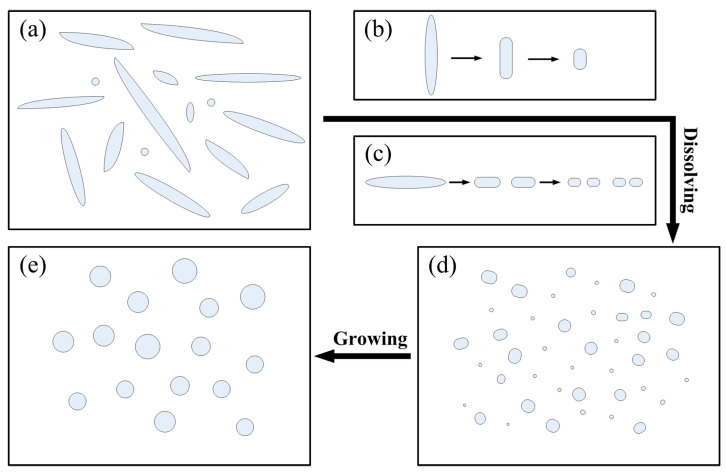
A sketch for the spheroidizing process of the second phase. When the second-phase in as-cast (**a**) is treated with pulsed current, the second-phase perpendicular to the direction of the current (**b**) and the second-phase parallel to the direction of the current (**c**) undergo different dissolution mechanisms. The dissolved second-phase continues to grow (**d**) and eventually becomes a spherical second-phase (**e**).

The spheroidizing process is presented in Figure 6. Due to the thermal and athermal effects of EPT, the strip-shaped S phases (Figure 6a) rapidly dissolve in the matrix via the combination effect of the two mechanisms, as shown in Figure 6b,c. On the other hand, when the EPT ends, the athermal effect of the current disappears; however, the thermal and compressive fields still exist, and these effects not only have an important effect on the dissolution process, but also play a positive role in the spheroidizing and growing process of the particles, implying that the size of the secondary phases can be controlled via changing the cooling speed after the EPT. According to the previously mentioned spheroidization and growth mechanisms, reducing the cooling rate allows the particles to undergo termination migration and growth processes during the cooling process. Hence, the EPT + WQ sample has a smaller-sized phase than the EPT + AQ sample, and the second phase of the EPT + AQ has a more regular shape than that of EPT + WQ.

## 4. Conclusions

In this paper, we achieved the rapid spheroidization process of second-phase (Al_2_CuMg) by using EPT technology. According to the characterization and simulation results, it can be proved that electropulsing can rapidly change the morphology of the second phase at room temperature in the as-cast 2024 al alloy through accelerated spheroidizing. This paper is informative for the study of second-phase morphological changes in aluminum alloys. Certainly, the diffusion process of alloying elements in 2024 aluminum alloy under electropulsing treatment is yet to be characterized and investigated in detail, which will be presented in our further work. The following are the conclusion in this study:

1. The second-phase (Al_2_CuMg) dissolution could be precisely controlled by EPT, and the morphology of the second phase was transformed from a rod-like shape to a spherical shape. For the different cooling methods, the EPT + AQ sample has a more regular shape than the EPT + WQ sample.
2. The athermal effect of the electropulsing treatment reduces the solid solution temperature of the second phase and promotes the diffusion of the alloying elements, thereby enabling the second phase to dissolve rapidly at a lower temperature.
3. The dissolution behavior of the second phase with two different orientations during electropulsing treatment was simulated using ANSYS software (2021 R1). For the second phase oriented perpendicular to the current, the current dissolves the tip of the second phase rapidly. In contrast, the second phase parallel to the current direction will start to dissolve from the middle, thus splitting the rod-like grains into dispersions with lower aspect ratios. The second phase of other orientations proceeds on the basis of the combined action of these two mechanisms.
4. The sphericity of the second phase in the EPT + AQ sample is higher than that of the EPT + WQ sample, which is a result from the spheroidization and growth of the second-phase grains due to the residual thermal and compression fields during the air cooling, which leads to a higher sphericity of the second phase in the samples of EPT + AQ.

## Data Availability

The data presented in this study are available on request from the corresponding author. The data are not publicly available due to technical or time limitations.
